# Differences in Epidural and Analgesic Use in Patients with Apparent Stage I Endometrial Cancer Treated by Open versus Laparoscopic Surgery: Results from the Randomised LACE Trial

**DOI:** 10.1155/2013/764329

**Published:** 2013-07-14

**Authors:** Jannah Baker, Monika Janda, David Belavy, Andreas Obermair

**Affiliations:** ^1^Queensland University of Technology, School of Public Health, Institute of Health and Biomedical Innovation, Brisbane, QLD 4059, Australia; ^2^University of Queensland, School of Medicine, Brisbane, QLD 4072, Australia; ^3^Queensland Centre for Gynaecological Cancer, Ned Hanlon Building, Butterfield St. Herston, Brisbane, QLD 4029, Australia

## Abstract

*Objectives*. We compared postoperative analgesic requirements between women with early stage endometrial cancer treated by total abdominal hysterectomy (TAH) or total laparoscopic hysterectomy (TLH). *Methods*. 760 patients with apparent stage I endometrial cancer were treated in the international, multicentre, prospective randomised trial (LACE) by TAH (*n* = 353) or TLH (*n* = 407) (2005–2010). Epidural, opioid, and nonopioid analgesic requirements were collected until ten months after surgery. *Results*. Baseline demographics and analgesic use were comparable between treatment arms. TAH patients were more likely to receive epidural analgesia than TLH patients (33% versus 0.5%, *P* < 0.001) during the early postoperative phase. Although opioid use was comparable in the TAH versus TLH groups during postoperative 0–2 days (99.7% versus 98.5%, *P* = 0.09), a significantly higher proportion of TAH patients required opioids 3–5 days (70% versus 22%, *P* < 0.0001), 6–14 days (35% versus 15%, *P* < 0.0001), and 15–60 days (15% versus 9%, *P* = 0.02) after surgery. Mean pain scores were significantly higher in the TAH versus TLH group one (2.48 versus 1.62, *P* < 0.0001) and four weeks (0.89 versus 0.63, *P* = 0.01) following surgery. *Conclusion*. Treatment of early stage endometrial cancer with TLH is associated with less frequent use of epidural, lower post-operative opioid requirements, and better pain scores than TAH.

## 1. Introduction

Endometrial cancer is the most common gynaecological cancer in developed countries and the current standard of treatment is hysterectomy and bilateral salpingectomy using an open abdominal surgical approach [1]. Three recent clinical trials have shown that a laparoscopic approach to surgery results in shorter hospital stay and fewer adverse events compared with open surgery [2–5] and two of these trials found better quality of life outcomes [3, 5]. Epidural and nonepidural opioid analgesia may be used for pain management following both, open or laparoscopic surgery, and guidelines are available for anaesthetic prescription and monitoring [6]. Although epidural analgesia may decrease the risk of cardiovascular and pulmonary complications in high-risk patients undergoing major surgery, it is invasive, costly, time consuming, and labour intensive [7]. There is developing evidence that other forms of regional analgesia may be more cost effective without sacrificing efficacy [7, 8].

Minimally invasive procedures have been found to be associated with smaller postoperative analgesia requirements compared with open surgery in patients treated for gynaecological cancers [9–12] and in ovarian metastasectomy from gastric cancer [13]. There is also evidence that total laparoscopic hysterectomy (TLH) offers benefits over vaginal hysterectomy in terms of reduced opioid and NSAID analgesic requirements following surgery [14].

Minimally invasive procedures for endometrial cancer, including total laparoscopic, laparoscopic-assisted vaginal, or robotic-assisted laparoscopic hysterectomy, are associated with significantly reduced postoperative analgesic use compared with open abdominal laparotomic hysterectomy [5, 15–18]; however, these studies only followed patients' perioperative analgesic requirements and there is a lack of randomised clinical trials comparing postoperative analgesic use between open and minimally invasive treatment arms during a more extended period of time after surgery and a lack of data from the Australian context.

This report examines differences in postoperative opioid and analgesic prescription between patients with apparent stage I endometrial cancer undergoing TLH or total abdominal hysterectomy (TAH) and outcomes of these patients up to ten months after surgery.

## 2. Methods

The LACE trial (laparoscopic approach to cancer of the endometrium) commenced recruitment in October 2005, and a total of 760 women with apparent stage I endometrial cancer were enrolled by June 2010 through one of 20 participating tertiary gynaecological oncology centres in Australia, New Zealand, Switzerland, and Hong Kong. The trial design and methodology, as well as QOL and AE outcomes, have been previously described [3, 4]. Women were eligible if they were 18 years or older and had a histologically confirmed endometrioid adenocarcinoma of the endometrium of any grade, an Eastern Cooperative Oncology Group (ECOG) score of less than two, and imaging studies (computed tomography (CT) of the abdomen and pelvis and chest radiograph or chest CT) suggesting the absence of extrauterine disease. Patients were excluded if they had histological cell type other than endometrioid on curettage, clinically advanced disease (stage II–IV) or bulky lymph nodes on imaging, uterine size greater than 10 weeks of gestation, estimated life expectancy of less than 6 months, medically unfit for surgery, or patient compliance or geographic proximity preventing adequate followup or if they are unfit to complete quality of life questionnaires. FIGO 2009 staging criteria were used.

Overall, 407 patients were randomised to TLH and 353 to TAH using block randomisation stratified by centre and grade of differentiation. Surgeons involved in the trial were all accredited gynaecological oncologists who had completed at least 20 TLHs, submitted video footage of a TLH, and performed a TLH live in the presence of a senior accredited surgeon. Seven patients withdrew before completing six weeks followup and were included in baseline and perioperative analyses but excluded from long-term comparison of analgesic use.

Details of medication use including medication name, dose, frequency, unit, route of administration, start date, and end date of prescription were recorded for each patient by the trial nurses at each hospital. Information was collected in detail during the perioperative period and then during the patients' postoperative one-week, four-weeks, three-month and six-month data clinical followup. Recorded start dates and end dates of analgesic prescription were used to categorise analgesic use into more distinct periods of time, up to a maximum record of 310 days (ten months) postoperatively.

Free-text entries of all medication names were scanned and classified into drug classes by one medical professional. All drugs classified as analgesics were further categorised by one of the authors (J. Baker) in consultation with anaesthetists, into opioid and nonopioid analgesia, and opioid analgesics further classified by route of administration (epidural, parenteral, or oral).

### 2.1. Statistical Analysis

Intention-to-treat analysis was used. Baseline demographic and clinical characteristics were compared between treatment arms using descriptive statistics. Descriptive statistics and chi-squared tests of heterogeneity were used to compare epidural, parenteral, or oral opioid requirements within two days of surgery and postoperative opioid and nonopioid analgesic requirements up to 10 months following surgery between treatment arms, based on comparisons of prescription start and end dates to date of surgery. Perioperative opioid use within two days of surgery was categorized by the most invasive route of administration for each patient. Long-term use of analgesics was explored by counting each patient in more than one analgesic category if they were prescribed more than one type of analgesic class. *t*-tests were used to compare postoperative pain scores between treatment arms.

## 3. Results

Within the TAH group (*N* = 353), 247 patients underwent a vertical midline incision, and 99 had a low abdominal transverse incisions. Among the TLH group, 24 patients needed to be converted and 11 of these underwent a vertical midline incision. Five patients randomised to TAH requested and received a TLH procedure (overall conversion rate 3.8%). Baseline characteristics and analgesic use were comparable between treatment arms, with 67% of patients overall not taking any analgesia, 29% taking nonopioid, and 3% taking opioid analgesia (Table 1).

Overall, 121/353 (34%) TAH and 2/407 (0.5%) TLH patients received pain relief through an epidural during the study period (*P* < 0.0001). At data collection two days after surgery, significantly more patients with TAH (116/353; 33%) had received an epidural compared with 2/407 (0.5%) of TLH patients (*P* < 0.0001, Table 2). Mean pain scores were significantly higher in the TAH versus TLH group at one week (2.48 versus 1.62, *P* < 0.0001), four weeks (0.89 versus 0.63, *P* = 0.01), and six months (0.45 versus 0.27, *P* = 0.04), but not at three months following surgery (Table 2).

During the first two postoperative days, although a similar proportion of patients in the TAH or TLH groups were prescribed opioid analgesia (99.7% versus 98.5%, *P* = 0.09) and NSAIDS (61% versus 60%, *P* = 0.7), a significantly higher proportion of TAH patients required Paracetamol (98% versus 95%, *P* = 0.03). At 3–5 days after surgery, significantly higher proportions of patients allocated to TAH required opioid analgesia (70% versus 22%, *P* < 0.0001), NSAIDs (38% versus 21%, *P* < 0.0001), and Paracetamol (91% versus 62%, *P* < 0.0001). This effect persisted at 6–14 days after surgery, with significantly higher proportions of patients allocated to TAH still requiring opioid analgesia (35% versus 15%, *P* < 0.0001), NSAIDs (24% versus 15%, *P* = 0.003), and Paracetamol (65% versus 46%, *P* < 0.0001). At 15–60 days after surgery, a significantly higher proportion in the TAH group still required opioid analgesia (15% versus 9%, *P* = 0.02) and Paracetamol (40% versus 28%, *P* = 0.0004), but a similar proportion in both treatment arms required NSAIDs (13% versus 9%, *P* = 0.2). Analgesic use was comparable between groups after 60 days after surgery (Table 3).

## 4. Discussion

Although patients undergoing TAH or TLH required narcotic analgesia for the first two days after surgery, those undergoing TLH recovered faster and fewer required analgesia by day three after surgery. This difference in analgesic requirements between the treatment groups persisted until after two months following surgery. Both the surgical approach and the epidural procedure could have contributed to these findings, as well as the greater prevalence of adverse surgical events observed among the TAH group [4]. Despite advances in the aftercare for patients with TAH, such as through fast-track surgical care [19], a significantly greater number of women require epidural analgesia for open abdominal compared to laparoscopic surgery for stage I endometrial cancer. As the LACE trial was unblinded, the anaesthetic prescription choices of the anaesthetists can be influenced by the planned procedure. As TAH patients require hospital care for a significantly longer time than TLH patients [4, 5, 20] providing an epidural conforms with recommendations to lessen the risk of prolonged immobilisation and the subsequent risk of thromboembolism in oncology patients [6, 21]. On the other hand, there is little evidence of decreased perioperative morbidity or mortality with epidural analgesia, particularly in the low to medium risk surgical population [7].

Our study is in agreement with a smaller Phase III trial conducted in the Netherlands which compared clinical and postoperative outcomes in 283 patients treated within 21 hospitals who were assigned to either laparoscopic or the standard procedure of open surgery for early stage endometrial cancer. Similar to the present study, this trial found the duration of pain after surgery to be significantly shorter for TLH versus TAH patients (median 3 (0–7) versus 5 days (0–7), *P* < 0.0001) [22]. However, this smaller trial followed analgesic outcomes perioperatively only, did not compare epidural use between treatment arms, did not distinguish between different classes of analgesia, and did not use or report pain score outcomes.

Our study findings are also similar to those of a prospective cohort study which found that women undergoing either laparoscopic or robotic surgery for endometrial cancer reported little need for opioid analgesia (45% did not require any analgesia, 34% required nonopioid analgesia, and only 21% required opioid analgesia) at 3-4 weeks after surgery [23]. In our study, during the comparable time period of 15–60 days after surgery, few patients undergoing TLH surgery had requirement for opioids (67% did not require analgesia, 28% required Paracetamol, 9% required NSAIDs, and 9% required opioid analgesia). Our study also supports findings from a prospective cohort study comparing minimally invasive surgery to open surgery [15]. This study involved 182 consecutive patients undergoing surgery for early endometrial cancer or endometrial hyperplasia with atypia and found that the patients receiving laparoscopically assisted vaginal hysterectomy (LAVH) (*N* = 74) had less need for analgesia than those receiving TAH (*N* = 108). Postoperatively, the laparotomy surgery group also had more frequent prolonged use of epidural analgesia than the LAVH group (72% versus 49%, *P* < 0.01). 

A retrospective analysis compared 181 consecutive patients with endometrial cancer undergoing open (*N* = 97) or minimally invasive staging hysterectomy (*N* = 84) including LAVH, TLH, or robotic-assisted laparoscopic hysterectomy using the da Vinci Surgical System, with or without lymphadenectomy [16]. This study found that in the open group, median surgery time was shorter (197 versus 288 minutes, *P* < 0.0001). Median narcotic (13 versus 43 mg morphine equivalents; *P* < 0.0001) and antiemetic (43% versus 25%; *P* = 0.01) needs, however, were lower for minimally invasive surgery already in the first 24 hours postoperatively.

A systematic review summarised the safety and efficacy of TLH versus open surgery in women with endometrial cancer and included 4 randomised clinical trials. This review specifically highlighted the reduced need for analgesia among women as one of the benefits of laparoscopic surgery [14, 17].

Besides the specific evidence related to endometrial cancer surgery to which the present study adds, there is also evidence that analgesic requirements and pain are reduced when minimally invasive surgery is applied to other gynaecological malignant conditions [11, 12] and are also less for women undergoing laparoscopic surgery for benign gynaecological conditions compared with an open surgical approach [10, 20]. For example, a review article examining surgical treatment for obese women with endometrial, cervical, and ovarian cancer found evidence that laparoscopic surgery was associated with less postoperative pain compared with open surgery [9].

Strengths of the present study include the fact that analgesic prescription can be compared between treatment arms within the context of a randomised clinical trial, a long follow-up period, distinction between different analgesic classes, inclusion of pain score comparisons, and the fact that a lower conversion rate than previous trials allows clearer inferences to be made regarding treatment arms. Limitations include the fact that the trial was unblinded, biasing decision-making for epidural and analgesic prescription.

In summary, the results of this study show that laparoscopic surgery for endometrial cancer is associated with less need for epidural and postoperative analgesic prescription compared with open surgery, saving on costs of analgesia and highlighting a further significant benefit to patients and the healthcare system of laparoscopic treatment over traditional open abdominal surgery. 

## Figures and Tables

**Table 1:** 

	TLH (*N* = 407)	TAH (*N* = 353)
Age in years, mean (SD)	63 (10)	63 (11)
BMI category^†^	*n* (%)	*n* (%)
Normal (18.50–24.99)	47 (12)	46 (14)
Overweight (25.00–29.99)	98 (25)	72 (21)
Obesity class I (30.00–34.99)	77 (20)	87 (26)
Obesity class II (35.00–39.99)	81 (21)	61 (18)
Obesity class III (≥40)	86 (22)	74 (22)
Education		
Completed ≤12 years of school	270 (70)	232 (70)
Completed >12 years of school	118 (30)	99 (30)
Employment		
Retired	170 (44)	134 (40)
Employed full time	55 (14)	42 (13)
Employed part time or casual	44 (11)	54 (16)
Other	119 (31)	101 (31)
Marital status		
Married or living together	243 (63)	212 (64)
Other	145 (37)	119 (36)
Private health insurance		
Yes	101 (26)	90 (27)
No	287 (74)	241 (73)
Income		
Less than AUS$40,000	261 (67)	207 (62)
AUS$40,000+	83 (21)	82 (25)
Not answered	44 (11)	42 (13)
Birth country		
Australia	249 (64)	219 (66)
Other	139 (36)	112 (34)
ECOG performance status		
0	352 (86)	303 (86)
1	55 (14)	50 (14)
Baseline analgesic use (pts categorised by strongest class taken)		
No analgesia	273 (67)	239 (68)
Nonopioid analgesia	120 (29)	103 (29)
Opioid analgesia	14 (3)	11 (3)

Data are number of patients (%). TLH: total laparoscopic hysterectomy. TAH: total abdominal hysterectomy. BMI: body mass index. ECOG: Eastern Cooperative Oncology Group. Numbers do not always add up to 760 because of missing demographic data. ^†^Based on WHO categories.

**Table 2:** Opioid use ≤2 days after surgery.

	TLH (*N* = 407)	TAH (*N* = 353)	*P* value
	*n* (%)	*n* (%)	
Route of postoperative opioid use*			
Epidural	2 (0.5)	116 (33)	
Parenteral	392 (96)	230 (65)	
Oral	5 (1)	2 (0.6)	
Nil	8 (2)	5 (1)	

	Mean (SD)	Mean (SD)	

Painscore			
Week 1	1.62 (2.01)	2.48 (2.13)	<0.0001
Week 4	0.63 (1.34)	0.89 (1.5)	0.01
Month 3	0.48 (1.39)	0.54 (1.26)	0.59
Month 6	0.27 (0.98)	0.45 (1.27)	0.04

*Patients categorised by the most invasive route of administration.

**Table 3:** Postoperative analgesic use, excluding 7 pts without 6-week followup*.

	TLH (*N* = 404)	TAH (*N* = 349)	*P* value
	*n* (%)	*n* (%)	
Analgesic classes 0–2 days after surgery			
Opioid	398 (98.5)	348 (99.7)	0.09
NSAID	242 (60)	214 (61)	0.69
Paracetamol	384 (95)	342 (98)	0.03
No analgesia	—	—	
Analgesic classes 3–5 days after surgery			
Opioid	87 (22)	246 (70)	<0.0001
NSAID	86 (21)	132 (38)	<0.0001
Paracetamol	252 (62)	317 (91)	<0.0001
No analgesia	125 (31)	14 (4)	
Analgesic classes 6–14 days after surgery			
Opioid	60 (15)	121 (35)	<0.0001
NSAID	61 (15)	82 (24)	0.0034
Paracetamol	187 (46)	227 (65)	<0.0001
No analgesia	190 (47)	86 (25)	
Analgesic classes 15–60 days after surgery			
Opioid	37 (9)	52 (15)	0.015
NSAID	37 (9)	44 (13)	0.24
Paracetamol	112 (28)	139 (40)	0.0004
No analgesia	271 (67)	180 (52)	
Analgesic classes 61–150 days after surgery			
Opioid	23 (6)	20 (6)	0.98
NSAID	20 (5)	20 (6)	0.63
Paracetamol	42 (10)	48 (14)	0.16
No analgesia	342 (85)	282 (81)	
Analgesic classes 151–310 days after surgery			
Opioid	13 (3)	10 (3)	0.78
NSAID	12 (3)	11 (3)	0.89
Paracetamol	19 (5)	16 (5)	0.94
No analgesia	369 (91)	319 (91)	

NSAID: nonsteroidal anti-inflammatory drugs.

*Patients counted more than once if taking more than one analgesic class.
